# Effect of experimental treatment on GAPDH mRNA expression as a housekeeping gene in human diploid fibroblasts

**DOI:** 10.1186/1471-2199-11-59

**Published:** 2010-08-14

**Authors:** Azalina Zainuddin, Kien Hui Chua, Norhazira Abdul Rahim, Suzana Makpol

**Affiliations:** 1 Department of Biochemistry, Faculty of Medicine, National University of Malaysia, Jalan Raja Muda Abdul Aziz, 50300 Kuala Lumpur, Malaysia; 2 Department of Physiology, Faculty of Medicine, National University of Malaysia, Jalan Raja Muda Abdul Aziz, 50300 Kuala Lumpur, Malaysia

## Abstract

**Background:**

Several genes have been used as housekeeping genes and choosing an appropriate reference gene is important for accurate quantitative RNA expression in real time RT-PCR technique. The expression levels of reference genes should remain constant between the cells of different tissues and under different experimental conditions. The purpose of this study was to determine the effect of different experimental treatments on the expression of glyceraldehyde 3-phosphate dehydrogenase (GAPDH) mRNA so that the reliability of GAPDH as reference gene for quantitative real time RT-PCR in human diploid fibroblasts (HDFs) can be validated. HDFs in 4 different treatment groups viz; young (passage 4), senescent (passage 30), H_2_O_2_-induced oxidative stress and γ-tocotrienol (GTT)-treated groups were harvested for total RNA extraction. Total RNA concentration and purity were determined prior to GAPDH mRNA quantification. Standard curve of GAPDH expression in serial diluted total RNA, melting curve analysis and agarose gel electrophoresis were used to determine the reliability of GAPDH as reference gene.

**Results:**

HDFs with different experimental treatments exhibited diverse cell morphology with different expression of senescence-associated β-galactosidase (SA β-gal) activity. However the expression level of GAPDH was consistent in all treatment groups.

**Conclusion:**

The study demonstrated that GAPDH is reliable as reference gene for quantitative gene expression analysis in HDFs. Therefore it can be used as housekeeping gene for quantitative real time RT-PCR technique in human diploid fibroblasts particularly in studying cellular senescence.

## Background

Cellular senescence has been most widely studied in fibroblast cells *in vitro *[1]. In many human cells, cellular senescence is characterized by several molecular and cytological markers, such as a large flat morphology, expression of a senescence-associated β-galactosidase activity (SA β-gal) [2], and altered gene expression [3]. Oxidative stress is a condition that arises when the production of reactive oxygen species (ROS) overwhelms the cellular antioxidant defences. Human diploid fibroblasts (HDFs) can develop a phenotype resembling senescence in response to oxidative stress. It has been reported that treating cells exogenously with certain hydrogen peroxide (H_2_O_2_) concentrations can trigger entry into a senescent-like state which is termed 'stress-induced premature senescence' (SIPS) [4].

The morphology of SIPS cells was found resembled of senescent cells; with gross enlargement and accumulation of granular cytoplasmic inclusions after 2 weeks exposure to low dose of H_2_O_2 _[5]. If oxidative stress or free radicals are at least partly responsible for lifespan and aging, it follows that antioxidant should prolong life and retard aging. Tocotrienol is one subclass of vitamin E that can be found abundantly in palm oil, rice bran oil, barley, corn, oats and wheat [6]. It acts effectively as an antioxidant because its hydrogen atom from the hydroxyl group on its chromanol ring can readily be donated to reduce free radicals, each has its own biological activity [7].

RNA differential displays [8] and the serial assessment of gene expression [9] have been applied to explore senescence-associated genes to gain an insight into the molecular mechanisms underlying senescence [10]. The study of the molecular mechanisms underlying senescence has shed light on central aspects of tumor development and has contributed to the research on organismal aging [11]. Quantification of transcription levels of genes plays a central role in the understanding of gene function [12]. Therefore, quantitative real time RT-PCR has become a popular means to assess mRNA expression level; due to its sensitivity, accuracy and ability to amplify mRNA signal [13]. The method allows detection of amplicon accumulation since it is performed using fluorogenic probes or intercalating dyes such as SYBR Green I, rather than by conventional end-point analysis [14]. However, it is essential to control for error between samples when measuring RNA expression. Therefore, in order to control for experimental variations in the amount of RNA used in each quantitative RT-PCR and batch-to-batch variations in PCR reagents, coincident measurement of so-called 'housekeeping' genes has been used for the normalization of target gene expression data [15].

Housekeeping genes or reference genes are essential endogenous regulatory genes that are involved in various processes in the cell, such as metabolism, cell structure, gene transcription, and homeostasis and are therefore constitutively expressed [16]. Choosing an appropriate reference gene is important for accurate quantitative RNA expression in real time RT-PCR technique. In using the relative quantitative RT-PCR method, the cycle thresholds of the genes of interest were compared to the housekeeping genes to determine relative changes in expression [16]. The expression levels of reference genes should remain constant between the cells of different tissues and under different experimental conditions [17]. If these requirements are not fulfilled then normalization to varying internal references can lead to increased 'noise' or erroneous results [18]. If the chosen housekeeping gene fluctuates randomly between samples, then small differences between genes of interest will be missed. Appropriate validation of internal references is therefore crucial to avoid misinterpretations of study findings [19].

Several genes have been used as housekeeping genes, including β-actin, β_2_-microglobulin, cyclooxygenase 1, glyceraldehyde 3-phosphate dehydrogenase (GAPDH), hypoxanthine phosphoribosyl transferase, porphobilinogen deaminase, and the transferring receptor [20]. The RNA encoding GAPDH is universally expressed. GAPDH catalyzes the oxidative phosphorylation of glyceraldehyde 3-phosphate to 1,3-bisphosphoglycerate during glycolysis as well as the reverse reaction in tissues involved in gluconeogenesis. GAPDH has also been implicated in other ubiquitous processes such as DNA replication and repair and apoptosis [21]. This gene has been used for real time comparative gene expression studies. However, recent research has demonstrated that the expression level of housekeeping genes may be altered due to differences in experimental treatment [22]. Therefore, it is important to validate the stability and elucidate the changes of the housekeeping genes between different experimental conditions. In order to determine the suitability of GAPDH as reference gene in senescent and antioxidant studies, total RNA from HDFs after different experimental treatments were used to rectify the consistency of GAPDH expression in different reaction conditions of human skin fibroblast cells senescent model.

## Results

### Morphology of human diploid fibroblast cells

**Figure 1:**
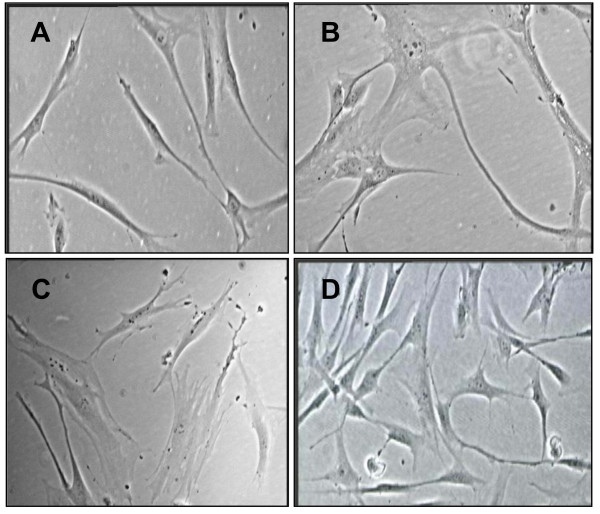
**Morphology of human diploid fibroblasts with different experimental treatments**. HDFs at young age, passage 4 (A) and senescent (passage 30) (B). The senescent cells showed increased cytoplasm volume and vacuoles, and loss its original fibroblastic shape by acquiring flattened feature. Cells with H_2_O_2_-induced oxidative stress showed similar morphological changes like senescent cells (C). The γ-tocotrienol-treated cells showed similar morphology to young cells (D). Micrographs are shown at ×200 magnification.

Young HDFs displayed spindle shape of adherent cells (Figure 1A). When the cells were at the end of replicative lifespan at passage 30, its morphology changed to enlarged cellular with flattening shape and all cells had higher ratio of cytoplasm:nucleus content (Figure 1B). The gradual loss of replicative potential results in reduced in cell yield. Similar senescence morphology was observed in HDFs induced with oxidative stress (Figure 1C). However, no morphological changes was observed in cells treated with γ-tocotrienol when compared to young HDFs (Figure 1D).

### Senescence-associated (SA) β-galactosidase expression

**Figure 2:**
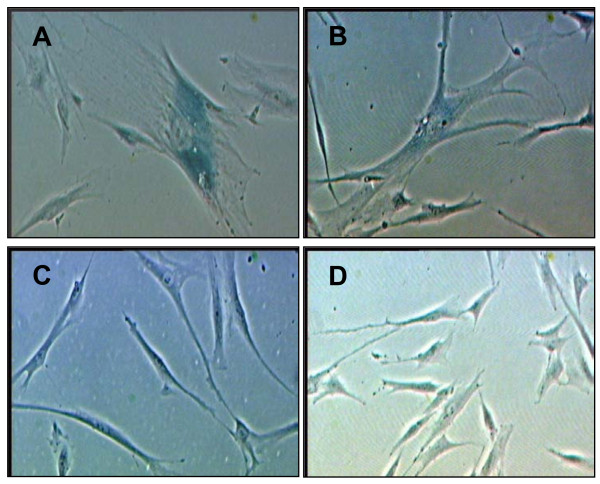
**Expression of senescence associated (SA) β-galactosidase with different experimental treatments**. Senescent HDFs, passage 30 (A); cells with H_2_O_2_-induced oxidative stress (B); HDFs at young age, passage 4 (C) and γ-tocotrienol-treated HDFs (D). Cells with blue staining indicated positive for β-galactosidase activity. Micrographs are shown at ×200 magnification.

Positive senescence-associated (SA) β-galactosidase staining was markedly increased in senescent cells at passage 30 (Figure 2A) and cells with H_2_O_2_-induced oxidative stress (Figure 2B). However, no positive staining was observed in young fibroblast cells (Figure 2C) and cells treated with γ-tocotrienol (Figure 2D).

### GAPDH mRNA expression

**Figure 3:**
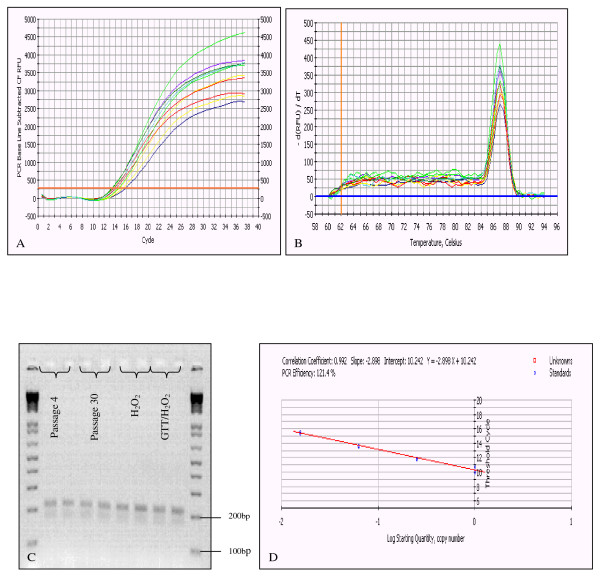
**GAPDH mRNA expression**. The Ct value showed the expression level of GAPDH was constant (within the acceptable range of 13-16) for the total RNA concentration in the range of 50-100 ng/μl (A). The melting curve demonstrated all PCR products had the specific melting temperature at 87°C (B). Agarose gel electrophoresis confirmed the size of all the PCR products generated was at 217 bp (C). The standard curve showed the threshold cycle (Ct) versus concentration of the total RNA from serial dilution (D).

Fluorescence was measured during each PCR cycle and the amount of fluorescence was proportional to the amount of the PCR product. The amplification graph showed that the Ct value for GAPDH of all treatment groups was within the range of 13-16 (Figure 3A) and was inversely proportional to the total RNA concentration isolated from HDFs with different experimental treatments (Table 1) indicating that GAPDH expression level was consistent with the concentration of total RNA (50-100 ng/μl). The melting curve showed the specificity of the GAPDH primer that has been designed, with each PCR product demonstrated one specific melting temperature (Figure 3B). Subsequently, the agarose gel electrophoresis which was performed to confirm the size of the PCR product in various treatment groups showed that the bands were specific, according to 217 bp (Figure 3C). The standard curve drawn with Ct value versus total RNA concentration showed the PCR reaction was at 121.4% of efficiency and correlation coefficient of 0.992 (Figure 3D).

**Table 1:** Total RNA concentration versus Ct value

Sample	Total RNA concentration	Ct value
Young	50.1 ng/μl	15.60
Senescent	56.8 ng/μl	14.65
H_2_O_2_-induced	73.5 ng/μl	13.55
γ-Tocotrienol/H_2_O_2_	73.6 ng/μl	13.70

## Discussion

Most type of primary normal cells did not proliferate indefinitely in culture. Instead, after a period of rapid proliferation, their division rate slows down and ultimately ceased altogether. Such cells became unresponsive to mitogenic stimuli yet can remain viable for extended periods of time. Upon entering the state of senescence, cells underwent a dramatic change in morphology; whereby their volume increases and they loss their original shape, acquiring a flattened cytoplasm [23]. These morphological changes were shown in our senescent HDFs and cells with H_2_O_2_-induced oxidative stress. This shift was accompanied by changes in nuclear structure, gene expression, protein processing, and metabolism [23]. Although there is limited study on the effects of tocotrienol and human life span, but it was found that tocotrienol administration reduces oxidative protein damage and extends the mean life span of *Caenorhabditis elegans*, one of the well established aging model [24]. These results strongly suggest that oxidative stress plays a role in bringing the changes in cellular function that occurs during aging.

In the present study, the RNA encoding GAPDH was ubiquitously expressed in all treatment groups. GAPDH is frequently used as an endogenous control for quantitative RT-PCR analysis because its expression is consistent at different time points and various experimental manipulations [25]. With the growing popularity of quantitative RT-PCR, one needs to justify whether the use of GAPDH as the specific internal standard RNA is appropriate [26]. As pointed out by Bustin (2002) [27], the ease and rapidity of which data was acquired by quantitative RT-PCR can easily create a false sense of objectivity. This assay used small amounts of RNA and therefore, is more prone to errors due to variation in RNA input [18].

The finding of this study indicated that GAPDH expression level was stable with total RNA concentration in the range of 50-100 ng/μl. This data suggested that GAPDH showed the suitability criteria as a housekeeping gene for human diploid fibroblasts particularly for studying cellular senescence. Both the H_2_O_2 _stress-induction and tocotrienol treatment did not essentially change the expression level of GAPDH. Moreover the real-time profile designed in this study with the highly specific primers used caused the reaction efficiency to be optimum with high correlation of the reaction. This finding was similar to Touchberry et al. (2006) [16] which reported that the average expression level of GAPDH among other genes in human skeletal muscle had the smallest different between old and young groups. Their findings also demonstrated that GAPDH was a stable housekeeping gene and appeared to be an effective gene for group comparisons between young and old populations. However, they also highlighted the limitation of GAPDH as housekeeping gene for comparison within groups due to its large variation from the mean. The high standard deviation scored within groups could be due to mRNA isolated from different individual with high biological variation. In comparison, our study used the same fibroblasts donors for all experiment groups. Thus, the biological variation that may affect GAPDH expression has been minimised.

The suitability of GAPDH as housekeeping gene has also been demonstrated in articular chondrocytes gene expression assessment in hypoxic condition [28]. GAPDH expression was not modulated in the chondrocytes by changes in oxygen tension. Studies on human glioblastoma and cancer cells of different origins for its molecular regulation under hypoxic condition also revealed GAPDH expression was not involved in the gene regulation [29,30]. In contrast, Zhong & Simons (1999) [31] showed GAPDH was increased in cancer cells under hypoxic conditions. GAPDH expression was correlated with the upregulation of hypoxia inducible factor-1 alpha. In another study, GAPDH was suggested as the target gene in the evaluation of amino-bisphosphophates effects on prostate and breast cancer cell lines [32]. The expression of GAPDH was significantly decreased in a dose-dependent manner following amino-bisphosphophates treatment to the cancer cells. GAPDH was also not suitable as housekeeping gene for cell lines under mitogens stimulation [33]. Therefore, it was highly recommended that the suitability of GAPDH as housekeeping gene be properly validated for each experiment to confirm that its expression was unaffected by different experimental conditions [34].

## Conclusion

This finding recommended that GAPDH should be the reference gene for future quantitative mRNA analysis involving human diploid fibroblasts particularly in studying cellular senescence.

## Methods

### Sample collection

This research has been approved by the National University of Malaysia Ethical Committee (Approval Project Code: FF-104-2007). Written consents were obtained from all subjects before tissue collection. Primary HDFs were derived from circumcision foreskins of 9-12 year-old boys.

### Cell culture

Skin samples were aseptically collected and rinsed several times with 75% alcohol and phosphate buffered saline containing 1% antibiotic-antimycotic (PAA, Austria) solution. After removing the epidermis, the dermis was cut into small pieces and transferred to 50 ml falcon tube (BD Bioscience, USA) containing 0.03% collagenase type I digestive buffer (Worthington Biochemical Corporation, USA). Dermis was digested in an incubator shaker at 37°C for 6 hours. The isolated HDFs were then centrifuged and rinsed with PBS twice. HDFs were cultured in Dulbecco's Modified Eagle Medium (DMEM, Flowlab, Australia) in T25 culture flasks containing 10% fetal bovine serum (FBS, PAA, Austria) and 1% antibiotic-antimycotic at 37°C in 5% CO_2 _humidified incubator. After 5 to 6 days, the cultured fibroblasts were harvested by trypsinization using 2.5% trypsin containing 0.03% of EDTA (Flowlab, Australia) and culture-expanded in T25 culture flasks. When the subcultures reached 80-90% confluency, serial passaging was done by trypsinization. The HDFs senescent model was consist of 4 different treatment groups; i) young (passage 4), ii) senescent (passage 30), iii) H_2_O_2_-induced oxidative stress (10 μM of H_2_O_2 _exposure for two weeks) and iv) γ-tocotrienol-treated group (20 μM treatment of γ-tocotrienol prior to H_2_O_2 _exposure). The morphology of the cells was assessed daily using inverted microscope.

### Morphological analysis and senescence-associated (SA) β-galactosidase staining

The molecular marker of cell-aging *in vitro *for HDFs (SA β-gal activity) was determined by senescent cells staining kit (Sigma, USA) according to the manufacturer's instruction. Senescent cells were stained blue after 4 h of incubation with β-galactosidase staining solution containing 5-bromo-4-chloro-3-indolyl-β-D-galactosidase (X-gal) at 37°C.

### Primer design

Primers for human GAPDH were designed from listed NIH GenBank database using Primer 3 software and blasted against GenBank database sequences for specificity confirmation. The sequence of GAPDH primers is shown in Table 2.

**Table 2:** Primer sequences of GAPDH

Gene	Sequence (5'-3')	Size of PCR product
GAPDH (Forward)	TCCCTGAGCTGAACGGGAAG	217
GAPDH (Reverse)	GGAGGAGTGGGTGTCGCTGT	

### RNA extraction

Total RNA from cultured HDFs in different treatment groups was extracted using TRI Reagent (Molecular Research Center, USA) according to the manufacturer's instruction. Polyacryl Carrier (Molecular Research Center, USA) was added in each extraction to precipitate the total RNA. Extracted RNA pellet was then washed with 75% ethanol and dried before dissolved in RNase and DNase free distilled water [35]. The yield and purity of the extracted total RNA were determined by Nanodrop (Thermo Scientific, USA) in triplicates. Total RNA concentration was then adjusted to 50-100 ng/μl before stored at -80°C immediately after extraction.

### Real time RT-PCR

Quantitative real-time RT-PCR reaction was carried out using 1 μl (50-100 ng/μl) total RNA as template; 1 ul of forward and reverse primers of GAPDH and iScript One-Step RT-PCR reagent with SYBR Green (Bio-Rad, USA). Reactions were conducted using Bio-Rad iCycler with reaction profile as follows; cDNA synthesis for 30 min at 50°C; pre-denaturation for 2 min at 94°C; PCR amplification for 38 cycles with 10 sec at 94°C and 30 sec at 61°C. This was followed by a melt curve analysis to determine the reaction specificity. Agarose gel electrophoresis was performed for confirmation of the size of PCR product.

### Standard curve

Total RNA extracted from various samples was serially diluted in nuclease-free water by 2 fold. The diluted total RNA was used as template for real-time RT-PCR and the experiment was conducted in duplicate for each sample. Master mix without total RNA was prepared for all reactions and 24 μl was aliquoted into each reaction tube and 1 μl of the diluted total RNA was then added in individually. Mean of Ct value was determined after the reaction in order to determine the linearity of the GAPDH expression level.

## Authors' contributions

AZ carried out the lab work and drafted the manuscript. KHC and NAR were involved in optimising the HDFs primary culture and revising the manuscript. SM was the Principal Investigator who designed the study and revised the manuscript. All authors read and approved the final manuscript.
